# The Many Faces of FKBP51

**DOI:** 10.3390/biom9010035

**Published:** 2019-01-21

**Authors:** Andreas Hähle, Stephanie Merz, Christian Meyners, Felix Hausch

**Affiliations:** Institute for Organic Chemistry and Biochemistry, Technische Universität Darmstadt, Alarich-Weiss-Strasse 4, D-64287 Darmstadt, Germany; haehle@drugdiscovery.chemie.tu-darmstadt.de (A.H.); merz@drugdiscovery.chemie.tu-darmstadt.de (S.M.); meyners@drugdiscovery.chemie.tu-darmstadt.de (C.M.)

**Keywords:** FKBP51, Hsp90, NF-κB, GR, glucocorticoids, FK506, SAFit

## Abstract

The FK506-binding protein 51 (FKBP51) has emerged as a key regulator of endocrine stress responses in mammals and as a potential therapeutic target for stress-related disorders (depression, post-traumatic stress disorder), metabolic disorders (obesity and diabetes) and chronic pain. Recently, FKBP51 has been implicated in several cellular pathways and numerous interacting protein partners have been reported. However, no consensus on the underlying molecular mechanisms has yet emerged. Here, we review the protein interaction partners reported for FKBP51, the proposed pathways involved, their relevance to FKBP51’s physiological function(s), the interplay with other FKBPs, and implications for the development of FKBP51-directed drugs.

## 1. Introduction

The FK506-binding protein 51 (FKBP51, encoded by the *FKBP5* gene) is an intracellular protein that can act as cochaperone in heat shock protein 90 (Hsp90) machinery. It displays peptidyl-prolyl isomerase activity and classifies as an immunophilin due to its tight binding to the immunosuppressants FK506 and rapamycin. It often acts in concert with its closest homolog, FKBP52, frequently in an antagonistic manner. The expression of FKBP51 is robustly induced by stress and (gluco)corticoid hormones. There is compelling evidence from mice and humans that FKBP51 plays an important role in stress endocrinology and glucocorticoid signaling. More recently, FKBP51 has been implicated in metabolism and in the regulation of chronic pain.

## 2. Structure of FKBP51

**Figure 1:**
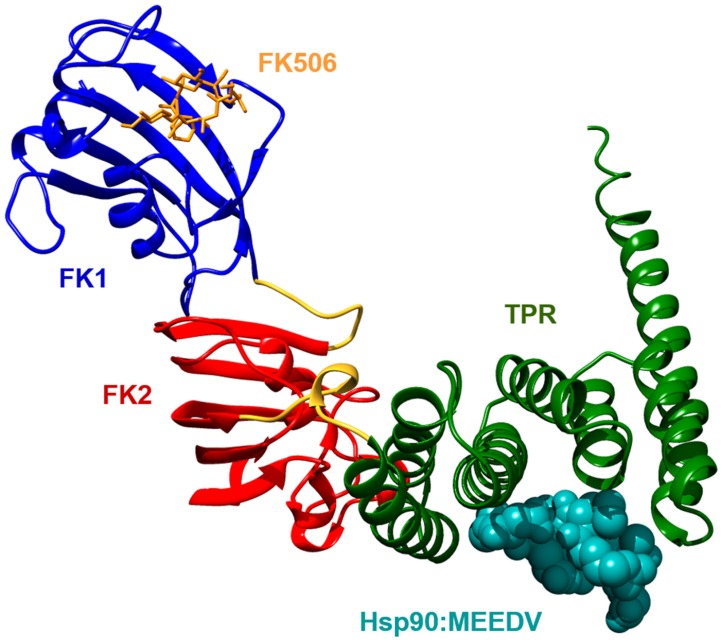
Full length FK506-binding protein 51 (FKBP51, PDB-ID: 1KT0) bound to the MEEDV motif derived from the heat shock protein 90 (Hsp90) C-terminus (PDB-ID: 5NJX). FK506 bound to the FK1 domain is superimposed from PDB-ID: 3O5R. TPR: tetratricopeptide repeat.

FKBP51 belongs to the larger FKBP proteins and consists of a FKBP-type peptidyl-prolyl *cis-trans* isomerase (PPIase) domain (called FK1), a FKBP-like domain (FK2) and a three-unit repeat of the tetratricopeptide repeat (TPR) domain (Figure 1) [1]. With a sequence identity of 60% and a similarity of 75%, FKBP51 is highly homologous to FKBP52. The domains of both proteins fold to a similar structure, but their relative orientation is different [2]. However, conclusions on functional differences of the overall architecture have to be handled with care as experiments indicate flexibility of the inter-domain linker regions, which allow the domains to adapt to different orientations relative to each other [3,4].

**Figure 2:**
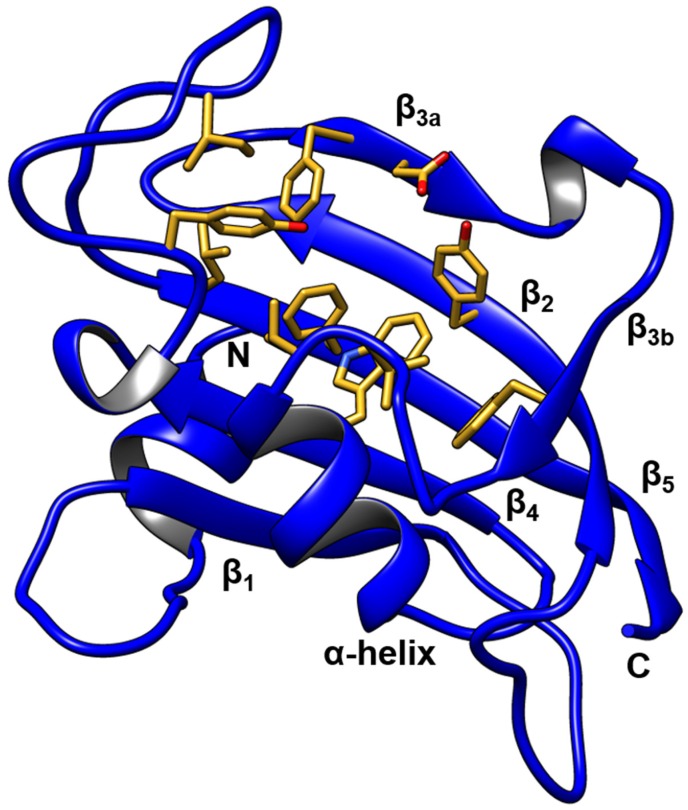
Structure of the FKBP51 FK1 domain. Conserved binding pocket amino acids are depicted in yellow (PDB-ID: 3O5E).

With 43 structures published, the N-terminal FK1 domain is structurally the best characterized element of FKBP51 (Figure 2). It shares 48% sequence identity with the archetypical PPIase domain of FKBP12 and adapts a similar structure compromising five antiparallel β-strands, which are curved around a central α-helix [1]. Similar to FKBP12, the FK1 domain of FKBP51 exhibits peptidyl-prolyl *cis-trans* isomerase activity, which can be inhibited by binding to the immunosuppressive drugs FK506 and rapamycin [1,5]. Both ligands bind to FKBP51 in a similar fashion as to FKBP12 and exploit similar key interactions comprising hydrogen bonds with tyrosine 113 and the backbone amide of isoleucine 87 [6,7]. Equivalent hydrogen bonds are observed for other FKBPs in complex with FK506 or rapamycin. In general, a structural comparison of the FK1 domains indicates a high conservation for key residues of the FK506 binding pocket. Regions of higher variability in proximity to the FK506 binding site are found for the residues of the tip of the β_4_-β_5_ interconnecting loop (Figure 2), the bulge intersecting the β_3_ strand as well as for residues facing away from the binding site in the β_2_ strand [7]. Despite the similar binding sites and modes, FK506 and rapamycin interact about 10 to 100 times stronger with the smaller FKBPs 12 and 12.6 compared to other FKBPs. A similar observation was made during the structure-guided optimization of pipecolate-derived non-immunosuppressive FK506 analogues [8]. However, especially the introduction of a conformationally constrained bicyclic core unit resulted in a series of high affinity ligands for human FKBPs as well as for microbial homologues [9,10].

**Figure 3:**
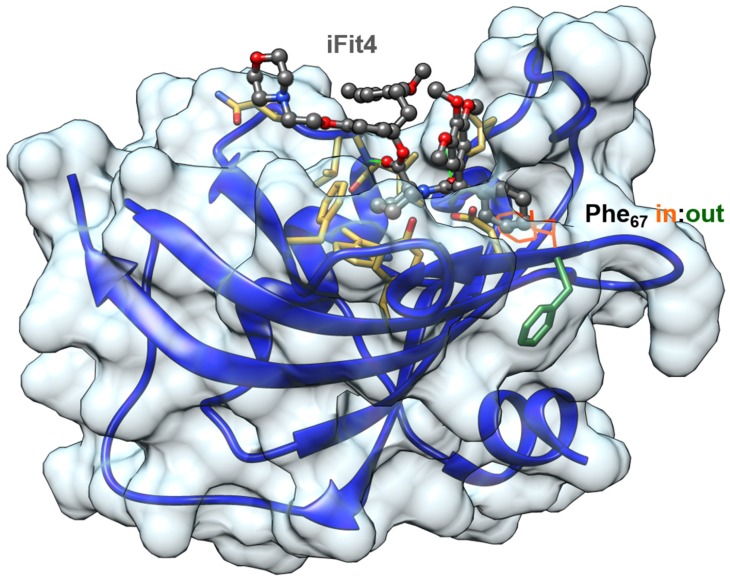
FK1 domain bound to the selective ligand iFit4 (PDB-ID: 4TW7). The Phe67-in state is superimposed from PDB-ID: 5OBK.

With a sequence identity of 71% (aa 41–140), the FK1 domains of FKBP51 and FKBP52 are nearly identical. Nevertheless, small differences in the β_2_ strand were found to be sufficient to develop selective FKBP51 ligands [11]. Interestingly, binding of the developed ligands of the iFit and SAFit series to FKBP51 causes a displacement of phenylalanine 67 from the binding site to an outward conformation, which is stabilized by lysine residues 58 and 60 (Figure 3). For FKBP52, the corresponding residues are threonine 58 and tryptophan 60 and indeed this structural difference impedes the necessary rearrangement for high affinity binding of iFit and SAFit ligands. While tryptophan 60 causes a sterical hindrance for the flip of phenylalanine 67, threonine 58 is engaged in a hydrogen bond with serine 69 interconnecting the β_3a_ and β_2_ strands, thereby suppressing intrinsic dynamics [11,12]. In general, FKBP51 shows a higher conformational plasticity in the β3 strand and the β_4_–β_5_ interconnecting loop than FKBP52, which can be suppressed by the introduction of FKBP52-like amino acid substitutions [12,13,14]. Interestingly, the generation of FKBP52-like FKBP51 variants not only prohibits the binding of FKBP51 selective ligands but furthermore also changes biochemical responses of FKBP51 indicating a connection of the intrinsic dynamics of FKBP51 to its biological functions [11,14,15].

In contrast to the FK1 domain, the function, structure and dynamics of the FK2 domain of FKBP51 are less well characterized and understood. In general, the FK2 domain adapts the typical FK fold albeit possessing several structural differences to the FK1 domain. Most strikingly, the bulge intersecting the β3 strand as well as the rapamycin binding site are absent [1,7]. The latter is due to an insertion of three residues in the β3-α-helix interconnecting loop. Although the deletion of these residues does not result in any measurable PPIase activity of the FK2 domain, it was suggested that they contribute to the binding of progesterone receptors, indicating that the FK2 domain possesses a scaffolding function and contributes to protein–protein interactions [1].

The C-terminal region of FKBP51 contains three repeats of the TPR motif, which fold into seven α-helices and mediate binding to the C-terminal MEEDV motif of Hsp90 [16]. They adapt a similar structure as the TPR domains of FKBP38, FKBP52 and other Hsp90 binding proteins like Hop or PP5 [1,2,16,17,18,19]. A comparison of co-crystallized structures indicates a similar binding mode of the MEEDV motif, and protein complex reconstitution experiments further suggest that FKBP51 competes with other TPR domains containing proteins for binding to Hsp90 [20]. Despite their structural similarity, TPR domain-containing proteins differ in their affinity for the MEEDV motif and, furthermore, their binding seems to be differently regulated. While the addition of small ubiquitin-like modifiers (SUMOylation) of FKBP51 seems to enhance Hsp90 binding, other interaction partners are sensitive to phosphorylation in proximity to the MEEDV motif [21,22]. Although the TPR domain as well as a flanking C-terminal motif are crucial for the binding of FKBP51 to Hsp90, recent NMR experiments suggest that the complex formation occurs via a large surface area which also involves the FK1 and FK2 domains [23,24]. Interestingly, the resulting complex is highly dynamic, providing evidence for the existence of intermediate states with either unbound FK1 or FK2 domains.

## 3. Human Genetics of FKBP51

The *FKBP5* gene encoding the FKBP51 protein consists of 13 exons located on chromosome 6 (6p21.31) [25,26]. The *FKBP5* gene contains several glucocorticoid response elements (GRE) in the promoter region and introns 2, 5 and 7. Upon binding of activated glucocorticoid receptors, these enhancers form three-dimensional chromatin loops and recruit RNA polymerase II to the transcription start site. Notably, numerous genetic variations in the *FKBP5* gene have been described, which are usually in linkage disequilibrium and are often represented by the single nucleotide polymorphisms rs3800373, rs9296158, or rs1360780. These polymorphisms have been functionally annotated to allow for stronger FKBP51 induction by glucocorticoids. For example, rs1360780 is located in intron 2 close to a functional GRE, and the rarer T allele was shown to lead to enhanced GR-mediated FKBP5 expression, likely by providing a new docking site for TATA-box binding proteins. The expression of FKBP51 is also epigenetically regulated. In carriers of the rs1360780 T allele, childhood abuse was associated with lower methylation of CpG sites near a functional GRE in intron 7 of the *FKBP5* gene [27]. Reduced methylation of this site was shown to allow for enhanced GR-induced transcription of FKBP5 [27]. 

In line with the role of FKBP51 in regulation of the hypothalamus–pituitary–adrenal axis (HPA, see below), the presence of the rs1360780 T allele or related haplotypes could be associated with HPA axis non-suppression after dexamethasone treatment and prolonged cortisol responses or self-reported anxiety symptoms after experimentally-induced psychosocial stress in healthy volunteers. The rs1360780 T allele was also associated with increased fear- or threat-induced hippocampal activation. Moreover, numerous studies have linked the rs1360780 T allele or related FKBP51-hyperinduction haplotypes to a higher risk for disorders such as major depressive disorders, post-traumatic stress disorder, suicidality, aggression, psychosis, and cognitive performance [25,26]. More recently, human genetic studies also reported associations of the FKBP5 hyper-inducing alleles with diabetes [28] and trauma-related chronic pain [29,30].

It has to be kept in mind that there are also negative findings on FKBP5 genotype associations and that genome-wide association meta-analyses studies have not detected significant main effects for the FKBP5 locus so far. Since many of the genetic associations described for FKBP5 seem to have depended on specific gene-environment interactions (e.g., interaction with stress or childhood trauma), genome-wide association studies so far may have been statistically underpowered to detect genetic interactions. Nonetheless, the combined human genetic studies strongly support the notion that FKBP51 plays a role in stress coping behaviour in humans and that it can be a risk factor for mood disorders.

## 4. Physiological Role of FKBP51 in Mammals

Under basal conditions, FKBP51 shows a pronounced expression in adipocytes and T cells. In humans, robust glucocorticoid-induced expression of FKBP51 has been observed in blood cells [31] and biopsies from skin [32] and adipose tissue [28]. In the rodent brain, basal FKBP51 expression is highest in the hippocampus, but strong induction of FKBP51 was observed in the amygdala and the paraventricular nucleus of the hypothalamus after a stress or glucocorticoid challenge [33,34]. Furthermore, FKBP51 was shown to be prominently upregulated in neurons in the dorsal horn in mice models of inflammatory pain [35].

Characterization of FKBP51-deficient mice revealed that FKBP51 plays a prominent role in the feedback control of the HPA axis, a key stress response system in mammals [36,37,38]. Furthermore, FKBP51^−/−^ -mice had an improved sleep profile [39] and enhanced glucose tolerance. They were resistant to diet-induced obesity [40,41] and protected from experimentally-induced forms of chronic pain [35,42] and glucocorticoid-induced skin hypoplasia [32]. The latter finding is surprising, since in the absence of FKBP51 (a suppressor of the glucocorticoid receptor), enhanced glucocorticoid-induced skin atrophy would have been expected. Indeed, no changes on a number of GR target genes could be detected in this study [32], pointing towards additional substrates of FKBP51 downstream of GR and FKBP51. Local viral overexpression of FKBP51 in airway epithelial cells reduced glucocorticoid responsiveness in an asthma model in mice, suggesting FKBP51 reduction as an anti-inflammatory (co)-treatment option for glucocorticoid-resistant asthma [43]. Importantly, no overt potentially adverse effects have been observed in FKBP51^−/−^ mice so far [44], suggesting FKBP51 to be a safe drug target.

Several pharmacological studies have been performed with SAFit2 (see also Section 6), the first ligand for FKBP51 with an acceptable selectivity and PK profile [11]. SAFit2 enhanced feedback inhibition of the hypothalamus–pituitary–adrenal axis [11], stress coping [11], and glucose tolerance [41]; was anxiolytic [33]; protected from weight gain [41]; and ameliorated mechanical hypersensitivity in inflammatory, neuropathic and chemotherapy-induced pain states [35,42]. When co-applied together with the antidepressant escitalopram, SAFit2 lowered the anxiolytic effect of escitalopram but dramatically improved stress-coping behavior [45].

## 5. FKBP Interaction Partners

### 5.1. Hsp90

The Hsp90 is a highly abundant and ubiquitously expressed chaperone in most mammalian cells. It is a central player in protein folding, stabilization, complex mediation, and degradation, with hundreds of client proteins, and, therefore, is involved in a plethora of cellular pathways and processes [46]. In early studies on steroid hormone receptors, FKBP51 and 52 (firstly entitled as p50 and p54) were successfully co-purified along with Hsp90 and the progesterone receptor [47] and later on were identified as immunophilins of the FKBP family [48]. FKBP51 and Hsp90 interact via their respective C-terminal domains. The FKBP51 TPR-domain binds the highly conserved MEEDV motif of Hsp90 [49]. The Hsp90 complex can be dissociated by the selective Hsp90 inhibitor geldanamycin, which also interrupts the attachment of immunophilins to the complex [50]. About nine TPR domain-containing proteins have been identified and confirmed to bind to Hsp90, such as the Hsp70-Hsp90 organizing protein (HOP), the serine/threonine protein phosphates 5 (PP5) and the E3 ubiquitin ligase CHIP (C terminus of HSC70-interacting protein), which is explicitly reviewed [46,51,52]. All of them share a 20 amino acid consensus sequence which is required for Hsp90 recognition [17,23], leading to a high competition for Hsp90 association which has been shown to be important for the regulation of steroid receptor signaling [53]. The differences in binding affinity and concentration of various TPR-domain proteins [54] reflect one layer to regulate the action of FKBP51 [55]. The dynamic association and dissociation of cochaperones, including PPIases, has been shown to be essential for the progression and fine-tuning of the conformational cycle of Hsp90 [46,56]. While it is clear that FKBP51 can associate with Hsp90, the involvement of Hsp90 as a mediating factor in the action of FKBP51 is often unclear for of many pathways described for FKBP51. 

### 5.2. Steroid Hormone Receptors

Both discovery and research motivation to study FKBP51 and other TPR-domain proteins is tightly connected to the investigation of steroid hormone receptors (SHR). These are the members of the SHR family: the androgen receptor (AR), the estrogen receptor (ER), the mineralcorticoid receptor (MR), the progesterone receptor (PR), and the glucocorticoid receptor (GR). Most SHR are clients of the Hsp90 chaperone machinery, which is essential for receptor maturation, hormone binding and translocation to the nucleus [57]. In a comprehensive study, Schülke et al. performed reporter gene assays and co-immunoprecipitations to investigate the impact of TPR proteins on SHRs [53]. Glucocorticoid and progesterone receptors were shown to be most sensitive towards the presence of TPR proteins, including FKBP51, while AR was only mildly affected and MR and ER were found to be largely unresponsive to TPR proteins. Both FKBP51 and its PPIase-deficient mutant are preferentially associated with PR and GR [58]. The *fkbp5* gene itself is inducible by glucocorticoids [59,60,61,62], progesterone [61,63,64,65] and androgenic hormones [66,67,68], leading to both elevated mRNA and protein levels. The *FKBP5* gene contains several glucocorticoid receptor response elements, and GR attachment mediates RNA polymerase II (PolII) loading and DNA methylation [69]. Soon it became clear that FKBP51 and especially the glucocorticoid receptor constitute an ultrashort negative feedback loop which is induced by steroid hormone receptors—elevated expression of FKBP51 reduces the transcriptional activity of those receptors. It was first shown in yeast that FKBP51 blocks the FKBP52-induced potentiation of the GR [70]. In mammalian cells, FKBP51 reduces GR reporter activity, which was connected in part to a reduced dynein binding of the receptor-chaperone-complex and, henceforth, a dampened translocation rate of the GR into the nucleus [71]. The PPIase activity seems not to be required for this mode of action since the FD67/68DV mutant that has no PPIase activity on peptide substrates retains GR inhibitory activity. However, amino acid 119 was found to be important in the different activity of FKBP51 and FKBP52. Pro119 as in FKBP52 supported receptor activation whereas L119 as in FKBP51 was inhibitory [15]. Mass spectrometry studies reveal similar complexes of GR/Hsp90/Hsp70/ATP with FKBP51 and FKBP52 as interchangeable factors in the early stages of complex formation. In those complexes, FKBP51 stabilizes the binding of the cochaperone p23, while FKBP52 leads to a release of p23, which primes the complex for nuclear translocation [20].

There might be other forms of GR and FKBP51 crosstalk. One study found that FKBP51 expression leads GR subform α-mediated adipogenesis [72]. Regulation by posttranslational modification has also been described for FKBP51. The attachment of SUMO at lysine 422, which was shown in vitro, was claimed to be important for GR inhibition in a reporter gene readout in hippocampal neuronal cells [22]. Recently, benztropine was claimed to diminish the inhibitory effect of FKBP51 on GR but the molecular mechanism of this remains to be elucidated [73]. The AR has largely been investigated in prostate cancer models. Contrary to GR, where FKBP51 has repeatedly been described as an inhibitory factor, two studies in 2010 reported an activation of AR by FKBP51 [74,75]. However, since these initial studies, no further confirmation of these findings has been published and the role of FKBP51 in prostate cancer biology remains controversial. The involvement of FKBP51 in steroid hormone signaling leads to numerous associations in physiological and pathological pathways involving a fine-tuned and cross-regulated interactome, which have been intensively studied and reviewed [76].

### 5.3. Akt and PHLPP

The kinase Akt serves as a central node to regulate various signaling pathways in growth and proliferation. Akt activity strongly depends on the phosphorylation of S473, which is thought to be regulated by the PH domain and leucine-rich repeat protein phosphatases (PHLPP). FKBP51 is believed to serve as a scaffolding protein that recruits PHLPP to Akt to facilitate dephosphorylation [77,78]. In support of this model, overexpression of FKBP51 was shown to reduce phosphorylation of S473 [77,78,79,80] and the Akt downstream targets glycogen synthase kinase 3 beta (GSK3β) and Forkhead box protein O1 (FOXO1). Conversely, FKBP51 knockdown or knockout led to an enhanced S473 phosphorlyation of Akt. Truncation studies suggest that the FK1 domain of FKBP51 is mainly required to recruit Akt [81] and the TPR domain to recruit PHLPP [77]. Further investigations revealed that the FK1 domain alone can bind Akt, as well as their respective PPIase mutants without being impacted by the presence of FKBP ligands [41,81], suggesting a competitive binding model for several FKBPs towards Akt. Not all studies could confirm the impact of FKBP51 overexpression on Akt [81]. Another study found that FKBP51 overexpression enhanced GSK3β phosphorylation in human embryonic kidney (HEK293) cells, which is counterintuitive to the suggested role of FKBP51 on Akt/PHLPP [82]. Furthermore, Beclin1, a trigger of autophagy and a substrate of Akt, was discussed as a mechanism linking FKBP51 levels to autophagy [79]. In the context of Akt regulation, the ubiquitin-specific peptidase 49 (USP49) could be co-purified with FKBP51. This deubiquitinase was claimed to stabilize FKBP51 and to enhance the dephosphorylation of Akt via PHLPP [78]. Moreover, the Akt–FKBP51 interaction was recently suggested to be regulated by acetylation of FKBP51 [80]. Six acetylation sites were reported, of which two are regulated by the deacetylase sirtuin 7 (SIRT7). Acetylation on the sites K28 and K155 were proposed to enhance FKBP51–Akt interaction, S473 dephosphorylation of Akt reduced phosphorylation of Akt downstream targets such as GSK3β. In 2017, the Akt substrate 160 (AS160) was coimmunoprecipitated along with FKBP51, Akt and PHLPP [41]. Its phosphorylation status could be disrupted by the selective FKBP51 ligand SAFit2, both in vitro and in vivo.

### 5.4. Nuclear Factor ‘Kappa-Light-Chain-Enhancer’ of Activated B-Cells (NF-κB)

FKBP51 was described as a regulator of NF-κB (nuclear factor binding near the κ light-chain in B-cells) signaling in different cell types. For this reason, FKBP51 has been suggested as a drug target for the treatment of NF-κB-mediated inflammation and cancer [43,83,84,85,86,87]. Nuclear factor-κB is a family of transcription factors affecting multiple cellular processes such as inflammation, proliferation, maturation, differentiation, survival, and apoptosis [88,89,90]. As a key factor, NF-κB regulates the innate and adaptive immune response [88,89,90]. In the last 15 years, several studies have aimed to elucidate the role of FKBP51 in NF-κB pathways and the underlying mechanisms [43,83,84,85,86,87,91,92,93,94].

Different FKBP51 interaction partners involved in NF-κB pathways have been identified. Among them, members of the IKK complex (inhibitor of nuclear factor kappa-B kinase subunits)—IKKα [43,83,85,92,94], IKKβ [43,85,94] and IKKγ [43,85,91,94]—belong to the most prominent interaction partners. The association of FKBP51 with IKK complex subunits was shown in various cell lines [43,83,85,91,92,94].

The role of the FKBP51 domains, FK1 (PPIase) and TPR, in the FKBP51/IKK interaction was controversially discussed. Specifically, the FK1 and TPR domain of FKBP51 both appeared to be required for its interaction with IKKγ in HEK293 cells [85]. Jiang et al. proposed that the FK1 domain is involved in the interaction with IKKα in human glioma cells (U87) [83]. The data indicated that the PPIase-inactive double-point mutation FD67DV of the FK1 domain reduced the interaction of FKBP51 with IKKα [83]. In contrast, Romano et al. did not observe any impact of the FD67/68DV mutation on either the FKBP51/IKKα/β or the IKKγ/IKKα/β interaction in HEK293 cells [85]. In accordance with these results, FK506, a FKBP51 isomerase inhibitor, did not affect the FKBP51/IKKα interaction [85]. Instead, a TPR domain mutant with diminished Hsp90 binding seemed to impair the interaction of IKKα/β with FKBP51 as well as with IKKγ in HEK293 cells [85].

FKBP51 was described as a TRAF (tumour necrosis factor receptor-associated factor) binding protein [85,91]. The findings suggested that FKBP51 interacts with TRAF2 in different cell lines as well as with TRAF3 and TRAF6 [91]. The TRAF2/FKBP51 interaction was investigated in depth by Romano et al. [85]. Based on co-immunoprecipitation assays in HEK293 cells, they hypothesized that the interaction of FKBP51 with TRAF2, probably mediated by the TPR domain, could promote the recruitment of IKKγ to TRAF2 [85]. Consequently, the formation and activation of the IKK complex could be positively affected by the FKBP51/TRAF2 interaction [85].

In addition, further potential interaction partners of FKBP51 such as the Hsp90 co-chaperone Cdc37 (cell division cycle control protein 37) [43,94], ReIA [93], TRAF5 [91], IRF3 (interferon regulatory factor 3) [91], IRF7 (interferon regulatory factor 7) [91], MAVS (mitochondrial antiviral signalling) protein [91] and TBK1 (TANK-binding kinase 1) [91] were found as a result of co-immunoprecipitation assays. Furthermore, IKKε [92], TAK1 (transforming growth factor β activated kinase1 = mitogen-activated protein kinase kinase kinase 7) [85,92] and MEKK1(mitogen-activated kinase kinase kinase 1) [92] have been described as potential FKBP51 interaction partners.

The involvement of Hsp90 in FKBP51-mediated NF-κB signaling is still under discussion. Hinz et al. indicated that Cdc37 could positively affect the FKBP51/IKKγ interaction in HeLa cells [94]. By contrast, Kästle et al. provided evidence for a Hsp90-independent interaction of FKBP51 with IKKβ and IKKγ in A549 cells [43]. Furthermore, Erlejman et al. did not find Hsp90 to participate in the suggested formation of a FKBP51/ReIA complex [93].

The effect of FKBP51 on NF-κB activation is also controversially discussed. On the one hand, various studies demonstrated that FKBP51 enhances NF-κB activation [85,86,92]. A reduction of NF-κB activation upon FKBP51 knockdown in HEK293 [92] or human melanoma (A375SM and FEMX-1) cells [86] was shown by reporter gene assays. Consistent with these findings, electrophoretic shift assay (EMSA) results provided evidence of an increased DNA/NF-κB interaction in nuclear fractions of glioma cells (U251 and U87) overexpressing FKBP51 [83]. In accordance with these data, EMSA results indicated that FKBP51 knockdown caused a decrease of nucleic NF-κB complexes in melanoma cells stimulated by irradiation [84] or TNF-α (tumor necrosis factor α) [85]. On the other hand, Erlejman et al. described the opposite effect of FKBP51 and concluded that NF-κB activation is regulated by the FKBP51/FKBP52 ratio [93]. Reporter gene assays implicated that FKBP51 impaired whereas FKBP52 enhanced PMA (phorbol 12-myristate 13-acetate) as well as TNF-α induced NF-κB activation in HEK293T cells overexpressing either FKBP51 or FKBP52, respectively [93]. Both effects seemed to be reversed by the expression of the corresponding TPR peptide [93]. A competition assay with FKBP51 and FKBP52 using the transcriptional activity of NF-κB as readout led to the conclusion that FKBP52 could act as an FKBP51 antagonist, reversing its inhibitory effect on NF-κB activation [93].

Another approach was to assess the impact of FKBP51 on the expression of NF-κB proteins as well as on their nuclear and cytoplasmic distribution [43,86]. Further investigations dealt with the FKBP51-mediated regulation of Inhibitor of NF-κB (IκB) protein levels [83,84,85,86,87].

More recently, Kästle et al. reported that FKBP51 knockdown in A549 cells resulted in a reduced nuclear protein level of p50 and p65 after IL-1β (interleukin 1β) stimulation [43]. In agreement with this study, an accumulation of NF-κB-p65 in the cytoplasm as well as a reduction of the nuclear level were observed in FKBP51 knockdown melanoma cells (A375SM and FEMX-1) [86].

Romano et al. suggested that FKBP51 depletion prevented the reduction of IκB-α as well as of IκB-β in irradiated melanoma cells [84]. These results are consistent with another study, which indicated a slight increase of the IκB-α protein level in FKBP51 knockdown cells (A172), as well as a slight decrease after overexpression of FKBP51 in U87 cells [83]. For that reason, Jiang et al. postulated that the degradation of IκB-α is positively controlled by FKBP51 expression in glioma cells [83]. In accordance with these findings, FKBP51 knockdown seemed to prevent IκB-α degradation induced by the chemotherapeutic drug Doxorubicin in human melanoma cells [87]. These findings are consistent with a lower phosphorylation grade of IκB-α observed in FKBP51 knockdown melanoma cells (A375SM and FEMX-1) [86]. Consistently, a kinase activity assay in melanoma cells (A375), using GST-IκB-α as substrate and TNF-α as stimulus, indicated a reduced phosphorylation of GST-IκB-α in FKBP51 knockdown cells compared with the corresponding controls [85]. The data suggested that FKBP51 inhibition impaired the kinase activity of the IKK complex [85]. Interestingly, compounds binding to the FKBP51FK1 domain—namely FK506, Rapamycin, SAFit1 and SAFit2—inhibited TNF-α induced IκB-α degradation in human melanoma cells (A375) [85]. The findings led to the conclusion that the PPIase domain could control IKK activity [85].

The effect of FKBP51 on NF-κB downstream signaling was assessed by analyzing the expression of NF-κB target genes in cells overexpressing or silencing FKBP51 [43,84,91,93]. Reporter gene assays indicated that PMA- or IL1β-induced IL6 (interleukin 6) expression could be downregulated by FKBP51 in BeWo cells [93]. An interesting study by Srivastava et al. provided evidence that IL-8 (interleukin 8) expression and release is controlled by FKBP51-mediated NF-κB signaling in melanoma cells (A375SM and FEMX-1) [86]. As a result of chromatin immunoprecipitation assays, silencing FKBP51 seemed to impair the interaction of NF-κB with the IL-8 promoter region [86]. Consistently, PCR, Western blot and ELISA (enzyme-linked immunosorbent assay) data showed that FKBP51 depletion decreased IL-8 expression and secretion [86]. Western blot analysis indicated that the expression of a constitutively active IKK-β mutant was able to bypass this effect [86]. By Western blot analysis, another study suggested that the upregulation of ICAM-1 (intracellular adhesion molecule 1) expression was inhibited in FKBP51 knockdown cells (A549) stimulated with IL-1β [43]. In addition, a reduced release of the chemokines CXCL-1 (GRO-1) and CXCL-2 (MIP-2a) was observed in FKBP51 knockdown cells (A549) stimulated with IL-1β [43]. Based on qPCR (quantitative polymerase chain reaction) analysis, Akiyama et al. suggested that FKBP51 knockdown in mouse embryonic fibroblasts (MEF) or fibroblast (L929) cells resulted in a decreased poly I:C-dependent expression of type I interferons, namely IFN-β and Isg15 [91]. A reduced transcriptional expression of IFN-β was displayed in FKBP51 knockdown MEF cells infected with Newcastle disease virus (NDV) as well [91].

To address the question whether FKBP51 acts as a scaffold protein affecting MAVS-mediated signaling, Akiyama et al. performed reporter gene assays based on the IFN-stimulated response element (ISRE) [91]. A decrease of the ISRE activation was observed in HEK293T cells overexpressing FKBP51 and either MAVS, the CARD domain of MDA-5 (melanoma differentiation-associated factor 5) or TBK1, supporting a scaffolding model [91]. 

Another interesting study by Romano et al. provided evidence that FKBP51 depletion could improve the therapeutic effect of ionizing radiation (Rx) in the treatment of malignant melanoma [84]. By performing Western blotting, they showed that the Rx-induced upregulation of xIAP (caspase inhibitor X linked inhibitor of apoptosis protein) expression could be suppressed by FKBP51 depletion in melanoma cells [84]. Flow cytometry data suggested that FKBP51 knockdown could trigger caspase-3 activation and consequently apoptosis in melanoma cells after irradiation [84].

In summary, there is compelling evidence that FKBP51 plays a role in the regulation of NF-κB signaling, with most studies suggesting a facilitating role of FKBP51. However, the underlying molecular mechanisms are controversially discussed and remain unclear. Intriguingly, various studies provided evidence that FKBP51 controls the IKK stability and thus the kinase activity. A pending question is whether these effects are tissue- or cell line-specific. In turn, these results underline the anti-inflammatory effect of FKBP51 suppression that could be exploited to improve the treatment of cancer—in particular melanoma—and inflammation-related diseases. A key question is to which extent currently available FKBP51 ligands can mimic a genetic reduction of FKBP51.

### 5.5. Other Interaction Partners

FKBP51 has been associated with a great number of other proteins. In a comprehensive interactome study addressing the Hsp90 complex and its cochaperones, numerous interaction partners of FKBP51 were described [55]. Among those findings are kinases such as the cyclin-dependent kinases (CDK1, 4, 9 and 11A) as well as kinases involved in cytoskeleton formation (Aurora kinase B, Fer). Furthermore, MYND-domain proteins, associated with transcriptional repression; minichromosome maintenance (MCM) complex proteins; helicase subunits and the argonaut proteins Ago1 and 2, essential components of the RNA-induced silencing complex (RISC), were identified as potential interactors. For CDKs, an antagonizing action mode of FKBP51 and 52 was described for their respective impact on DNA methylation [95]. Both immunophillins were co-purified with the CDK5 and its downstream target DNA methyltransferase 1 (DNMT1) [82,95]. Higher FKBP51 expression was linked to decreased DNMT1 phosphorylation levels and reduced methylation levels of the brain-derived neurotrophic factor locus in human blood samples. Accordingly, FKBP52 was suggested to exhibit the opposite effects [95]. In addition, FKBP51 was also found to bind to CDK4, a known oncogene. FKBP51 knockdown led to decreased CDK4 expression [96].

Although the evidence of FKBP51 being involved in the cytoskeletal processes accumulates, an overall model for its action mode is still lacking. The microtubule-forming monomer Tau is linked to plague formation in Alzheimer’s Disease (AD). In 2010, it was found that the overexpression of FKBP51 increases Tau concentration and FKBP51 knockdown reduces it. Both proteins could be co-purified. FKBP51 seemed to protect Tau from ubiquitination potentially acting as a chaperone, since Hsp90 was found in this complex as well. An active PPIase pocket was required for binding Tau [97]. In the same year, FKBP51 and its partial counter player FKBP52 were linked to another process involving microtubule arrangements. FKBP51 dampens while FKBP52 enhances neurite outgrowth during neuronal differentiation [11,98], which requires elevated expression of cytoskeletal proteins. The anti-neuritotrophic activity of FKBP51 could be blocked by FKBP51 ligands which increase neurite outgrowth in neuroblastoma cell lines and in primary embryonic neurons [11,99,100].

Interestingly, the connection of FKBP51 and microtubule formation via tau dephosphorylation was recently proposed to be protein phosphatase 5 (PP5C)-mediated, a TPR-domain containing phosphatase [101]. Furthermore, expression levels of both proteins affect the store-operated calcium entry current in pulmonary artery endothelial cells and HEK293 cells. Additionally, store-operated channels have been reported to be desensitized by FKBP51. FKBP52 was shown to antagonize this effect [102]. Immunophilins, such as FKBPs 52, 12, 25, and 38 have generally been associated with calcium ion channels especially of the TRPC family, and effects of high concentrations of FK506 on calcium flux have been reported [103,104,105,106]. 

Yet another interaction of FKBPs with rather unknown impact is described with the E3-ubiquitin ligase regulator Glomulin [107]. Mutations in the Glomulin gene lead to Glomuvenous malformation [108,109,110]. FKBP12 and FKBP52 have been shown to bind Glomulin. Point mutants and truncation mutants lacking Prolin219 of Glomulin showed significantly reduced β-galactosidase activity in a yeast two-hybrid screen [111]. The function of this interaction remains cryptic.

This RISC complex protein shows elevated expression with increased FKBP51 and 52 levels in mural embryonal stem cells and is less abundant if one or both immunophilins are knocked down. Interestingly, less Ago2 can be found if the cells are treated with FK506 [78,112].

**Figure 4:**
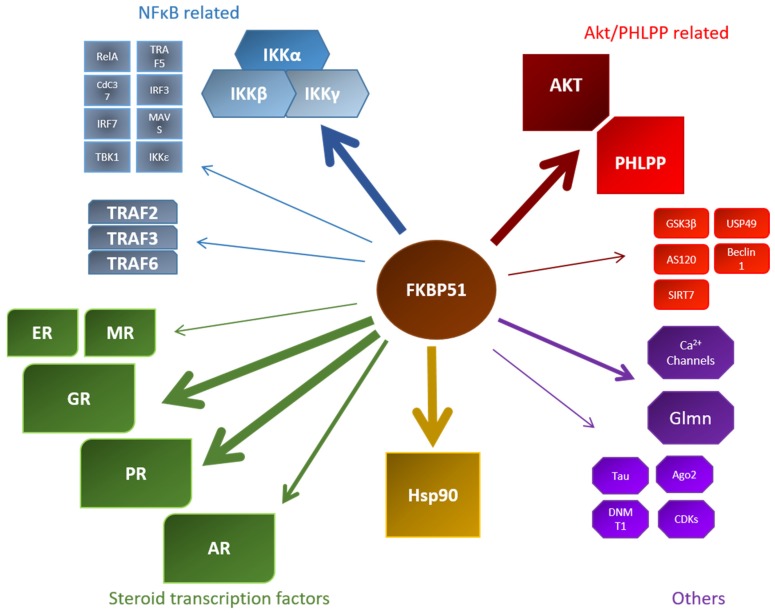
Overview of known FKBP51 interactors within their respective field of discovery. Stronger arrows indicate a larger data set of this specific interaction. MR: mineralocorticoid receptor; CDK: cyclin-dependent kinase; ER: estrogen receptor NFκB: nuclear factor binding near the κ light-chain in B-cells; USP: ubiquitin-specific protease

The described interactions of FKBP51 are summarized in Table 1. A graphical overview is given in Figure 4.

## 6. Implications for Drug Discovery

As an intracellular protein, only small molecule interventions are applicable to FKBP51, which in addition have to be brain-permeable for a potential treatment of depression or chronic pain. To exert a possible therapeutic effect, FKBP51 inhibitors likely have to be applied over extended times, ideally in an oral way. FBKP51-directed drugs will thus have to have very sophisticated pharmacokinetic parameters and a high-end safety profile.

**Figure 5:**
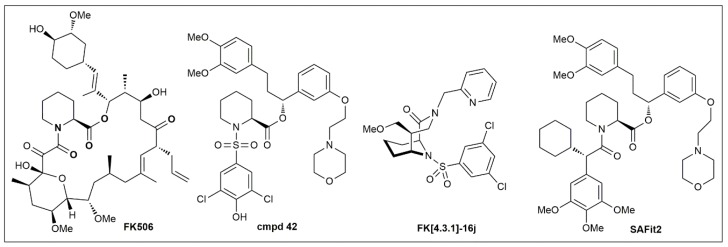
Chemical structures of important FKBP51 ligands.

To develop pharmacological tools for FKBP51, our group started from FK506 as the only available chemical starting point (Figure 5). Based on co-crystal structures [4,7] and a fluorescence-based competitive binding assay [5], we performed a first systematic analysis of the binding requirements for the FK506-binding site of FKBP51 [8,113], which led to simplified pipecolate scaffolds such as Cmpd 42 [8,113] (Figure 5). To regain binding energy, we rigidified the pipecolate core [9,100,114], leading to highly potent FKBP ligands such as FK[4.3.1]-16j [10]. However, none of these compounds were able to differentiate between FKBP51 and FKBP52, which in light of the opposing role of these two proteins seems to be an absolutely stringent requirement for FKBP51 pharmacology.

The problem of FKBP51/FKBP52 selectivity was solved when we discovered that FKBP51 can adopt a new conformation that is unavailable to FKBP52 (Figure 3) [11]. This led to the SAFit class of ligands, which consistently display selectivities up to 10,000-fold for FKBP51 over FKBP52 [99,115]. SAFit2 (Figure 5), currently the most advanced and best characterized FKBP51 ligand, has shown promising effects in numerous animal models (see Section 4). However, the pharmacokinetic properties of SAFit2 such as solubility, metabolic stability and oral bioavailability of SAFit2 are far away from a desired central nervous system drug profile. Towards optimizing SAFit2 into a clinical candidate, substantial structural modifications may be necessary, most importantly the reduction of molecular weight (currently 802 g/mol).

An important mechanistic pending question for future FKBP51 drug discovery is the biological relevance of the FK506-binding pocket. This position is currently the only site in FKBP51 that can be targeted with small drug-like molecules. It is increasingly becoming clear that some biological functions of FKBP51 do not involve this FK506-binding site but rather depend on scaffolding functions of other domains [43,81,116].

## 7. Conclusions

Substantial evidence from transgenic mice and human genetics suggest that FKBP51 plays a key role in stress biology, metabolism and pain signaling. Towards leveraging FKBP51 as a target for diseases such as depression, obesity or chronic pain, the unclear molecular mechanism of how FKBP51 affects cellular processes has become a major limitation. Numerous interaction partners have been described for FKBP51, many of which remain to be independently confirmed. For those interaction partners of FKBP51 that were studied by several groups, conflicting results have been obtained and no clear consensus has emerged. FKBP51 might be a very sticky protein and many of the interactions that can be detected by co-immunoprecipitation might not be functionally relevant. Notably, with the exception of binding to Hsp90, none of the suggested molecular functions of FKBP51 have been reconstituted with purified proteins in a defined biochemical setup. Rigorous biochemical analysis of FKBP51’s molecular mode of actions is of the utmost importance in the future.

## Figures and Tables

**Table 1:** Overview of FKBP51 interaction modes.

Interaction Partner	FK1 Domain Dependency	TPR Domain Dependency	Remarks
Hsp90	a [49]	d, e [49]	FKBP52 competitive [53]
GR		via Hsp90 [58]	SUMOylation dependent [22] FKBP52 competitive [70]
PR	b [58]	d [58]	
AR	c [74,75]	[74]	
Akt	a [77]	e [81]	Deubiquitination [78] and acetylation dependent [80]
PHLPP	a [77]	d [77]	
AS160	c [41]		
GSK3β	a [82]	d [82]	
DNMT1	b [95]		FKBP52 competitive [95]
SIRT7	[80]		
IKKα	b,c [83,85]	e [85]	
IKKβ	b [85]	e [85]	
IKKγ	b [85]	e [85]	
TRAF2	b [85]	d,e [85]	
Tau	b [97]	[97]	
Ago	c [112]		

Color indicates if the interaction but not the subsequent function is mediated by FK1 or tetratricopeptide repeat (TPR) domain. Red: not required, Green: required, Yellow: Controversial. (a) Investigated with FK1 domain deletion mutants; (b) Investigated with peptidyl-prolyl cis-trans isomerase (PPIase) point mutants; (c) PPIase binding drug sensitive; (d) Shown with TPR truncation mutant; (e) Shown with TPR point mutants. GR: glucocorticoid receptor; PR: progesterone receptor; AR: androgen receptor; PHLPP: PH domain and leucine rich repeat protein phosphatase; AS160: Akt substrate 160; GSK3β: glycogen synthase kinase 3 beta; DNMT1: DNA methyltransferase 1; SIRT7: sirtuin7; IKK: inhibitor of nuclear factor kappa-B kinase subunits; TRAF2: TNF receptor-associated factor 2; SUMO: small ubiquitin-like modifiers.
